# Addressing ethical challenges in the reporting of race and ethnicity population descriptors in human neuroscience research

**DOI:** 10.3389/fnimg.2026.1744569

**Published:** 2026-07-01

**Authors:** Lauren Lang, Julianna Gerold, Eric Little, Craig McFarland, Beri Tawe, Rishab Kumar Jain, Francis X. Shen

**Affiliations:** 1 Department of Biology, Emory University, Atlanta, GA, United States; 2 Massachusetts General Hospital, Athinoula A. Martinos Center for Biomedical Imaging, Charlestown, MA, United States; 3 Harvard College, Cambridge, MA, United States; 4 Brown University, Providence, RI, United States; 5 Neurotech Justice Accelerator at MGB, a Dana Foundation Center for Neuroscience & Society, Harvard Medical School Center for Bioethics, MGH Center for Law, Brain & Behavior, University of Minnesota Law School & Graduate Program in Neuroscience, Minneapolis, MN, United States

**Keywords:** ethnicity, race, journal publishing, neuroimaging, population descriptor, ethics

## Abstract

Increased racial and ethnic diversity in population neuroscience research is widely understood to facilitate better identification of subgroup effects and more generalizable findings. Consistency in reporting race and ethnicity population descriptor variables would allow the research community to better assess progress toward more representative datasets. One important lever for ensuring robust and consistent reporting of population descriptors are journal guidelines, and this review of current guidelines finds that there are opportunities for neuroscience journals to strengthen scientific rigor by more clearly delineating expectations with respect to reporting and operationalizing race and ethnicity population descriptors.

## Addressing ethical challenges in the utilization of race and ethnicity population descriptors in human neuroscience research

A population descriptor is a variable or category used for “describing or distinguishing people from each other based on perceived or actual differences” (National Human Genome Research Institute, 2024). In this article, we focus on race and ethnicity. There is growing understanding that race is a sociopolitical construct that does not map onto genetic ancestry, yet race and ethnicity are often used as a proxies for genetic ancestry and exposure to social determinants of health (National Academies of Sciences, Engineering, and Medicine, 2025).

Scholars have debated the merits of continuing to measure, utilize, and report race and ethnicity population descriptors in biomedical research. Arguments to abandon or modify the use of population descriptors distill into concerns of precision and morality. When race or ethnicity are used to approximate social determinants of health, researchers cannot precisely identify the mechanisms underlying observed effects. Moreover, attributing differential health outcomes to race places the onus of disease on the individual, obscuring systemic causes of health inequity and reifying the false notion of biological race.

Counterarguments propose that mindful use of these variables can promote representativeness in research, increase generalizability, and ensure scientific findings remain accessible and interpretable to the populations to which they may apply. Together, this debate underscores the need for a nuanced approach that neither treats race and ethnicity as biological proxies nor dismisses their ethical relevance in health research.

This on-going debate has shaped new policies governing how journals report on participants’ race and ethnicity, and how biobanks utilize these categories (Kaplan and Bennett, 2003; Lee, 2015; Routen et al., 2021; Smart et al., 2008; Tutton, 2009; Winker, 2006). These issues remain unresolved, however, and at the core of the debate is whether including race as a variable in empirical research perpetuates racist assumptions about biologically-based differences, or whether it instead provides a foundation for addressing racial disparities in health (Lee, 2009). For example, the 2022 National Academies Report on Improving Representation in Clinical Trials and Research provides several examples of how recognizing the distinct medical needs of various groups has contributed to reductions in health disparities (National Academies of Sciences, Engineering, and Medicine, 2022).

While these debates emerged early in fields like public health, where race has long been included in empirical scholarship (LaVeist, 1996), this debate is relatively new to neuroscience. Contemporary neuroscience research has historically neither included race/ethnicity in its empirical models, nor reported those variables in summary statistics (Goldfarb and Brown, 2022; Sterling et al., 2022). The advent of population neuroscience necessitates revisiting race and ethnicity variables in human neuroimaging research. A landmark study by Marek et al. highlights the importance of diversity in this context, revealing that although the median sample size of neuroimaging studies is about 25 participants, achieving reproducibility—and thus generalizability—in brain-wide association studies requires samples sizes in the thousands (Marek et al., 2022). Small or poorly sampled datasets inflate effect sizes due to individual variability and inadequate control of covariates. Recent large-scale neuroimaging datasets and biobanks now enable more robust examination of how demographic, environmental, and structural factors shape brain-behavior relationships, while also introducing new methodological and ethical challenges. As larger sample sizes inherent in population neuroimaging invite comparisons across individuals and groupings of those individuals, it is essential to consider how demographic variables are defined, operationalized, utilized in analysis, and reported.

We note at the outset that we do not take a stance in this essay on *whether* race and ethnicity should be utilized in a particular study. In a separate, related work we are collaborating with a larger, interdisciplinary working group supported by an NIH BRAIN Initiative Neuroethics grant (R01MH134144) that is tasked with addressing that question. Here, we focus on two related questions, one empirical and one normative:

- The empirical question: What is current neuroscience journal guidance with respect to reporting race and ethnicity population descriptors, and what variation exists across journals?
- The normative question: *Should* there be consistent guidance on reporting these variables, and if so, what should that guidance be?

## Methods

To answer the empirical question, we examined the 25 highest impact factor (as of May 2024) journals included in the “Neurosciences” category of the Observatory of International Research rankings (Observatory of International Research, 2024). We excluded two journals, *Molecular Neurodegeneration* and *Sleep Medicine Reviews* due to insufficient inclusion of human neuroimaging papers and insufficient information posted on their website, respectively. We included four additional journals—*Human Brain Mapping, Science, Nature,* and *Oxford Open Neurosciences*—where important human neuroimaging research studies are published.

From June 28, 2024 to July 10, 2024, we examined each journal’s website to review the guidance provided by journals to authors on how to report population descriptors. The senior editors of each journal^1^ were also contacted via email regarding their journal’s policies for reporting demographic information. We asked: “Could you please provide information on your journal’s current standards, policies, or guidelines for reporting demographic data in human neuroscience studies?” If there was no formal policy, we asked that the editors share with us the journal’s informal norms. Of the 25 journals contacted, 19 responded^2^.

In this article we focus exclusively on guidelines specific to reporting race and ethnicity. We recognize that journals comply with other ethical publishing guidelines, e.g., the Declaration of Helsinki and the Committee on Publication Ethics (COPE)^3^ (Wager, 2012; World Medical Association, 2013). The former promotes diverse sample recruitment while the latter promotes ethical conduct of research. However, neither ethical guideline states whether or how to report the demographics of research participants (World Medical Association, 2013).

## Results

### Lack of standardized guidelines

Our analysis revealed a lack of standardized guidelines across journals, as well as a lack of prescriptive instructions for authors. Our survey revealed five important conclusions:

1. Some journals offer no guidance at all. 21 out of 27 (78%) journals provide authors with at least some guidance on the reporting of race and ethnicity population descriptor variables. Four of these journals (15%) offer guidelines limited exclusively to clinical trials, and not for all studies involving human participants. This leaves 22% of journals offering no guidance at all.
2. There is a lack of standardized guidelines adopted across the journals, with six different guidelines adopted across the 27 journals. The three most common guidelines were ICMJE (or ICMJE for Biomedical Research), *Nature*, and AMA Manual of Style.
3. Most guidelines do not state how authors should define and operationalize racial and ethnic categories. Instead, they typically require that reported categories be defined and that any decision to omit race and/or ethnicity be explained. More detailed guidelines, such as those from *Nature* and the AMA, offer guidance on terminology and reporting practices. However, only one journal, Human Brain Mapping, explicitly states a preference regarding how demographic categories should be reported, favoring ethnicity over race.
4. Seven out of 27 (26%) of journals abided by one of two more comprehensive guidelines, *Nature* or AMA Manual of Style. While these more comprehensive guidelines do not offer instruction on how to define racial categories, they do require authors to report demographic variables more thoughtfully. Five journals follow *Nature*’s guidelines on reporting race and ethnicity in human subjects research. These guidelines call for reporting race and ethnicity (or justifying omissions), using inclusive and respectful language, considering genetic ancestry, and recognizing that manuscripts may be revised or rejected for racism or harmful content. Two journals follow the *AMA Manual of Style*, which emphasizes in its 2024 update that race, ethnicity, and geographic origin are not valid proxies for genetic ancestry. AMA guidelines also require authors to use inclusive and specific language, pay attention to privacy and ethics, use nuanced categorization, and acknowledge limitations in study generalizability.
5. Not all editors were aware of their journal’s policies. Correspondence with one senior editor revealed that despite publishing author guidelines on their website, they indicated that, “We have no specific policies on the description [of race and ethnicity demographic variables].”

Given that editorial staff are not always fully aware of their published guidelines, we wondered how this impacts author compliance.

### Lack of author adherence

After reviewing the guidelines, we sought to assess the publications’ reporting of race, ethnicity, and ancestry demographic data and adherence to corresponding journal guidelines (Biological Psychiatry, 2023). Recognizing that author adherence with journal policies may vary, we conducted a pilot analysis of recent publications in one journal, *Biological Psychiatry*. We selected this journal as an illustrative example because to its great credit, *Biological Psychiatry* stands out as a leader in the field for being one of the two journals that adopted the AMA guidelines (which require reporting of race and ethnicity, or an explanation for why race and ethnicity were not used). We reviewed all human subjects research articles published in *Biological Psychiatry* over a six month period, from February 1, 2024 through August 15, 2024.

We found that 30 out of 61 articles (49%) reported on participant’s race, ethnicity, and/or ancestry. Only a single article complied with all AMA guidelines on reporting race and ethnicity, doing so largely because its homogenous sample rendered many reporting guidelines inapplicable. By noting and considering the implications of a sample with limited diversity, the authors met all AMA requirements.

We can only speculate as to why author adherence was about 50%. One likely reason is that these policies are relatively new, and when investigators were being trained on how to write research articles, instruction on how to report race and ethnicity was not a priority. A second reason may be that journals do not always enforce these guidelines.

## Discussion

This Perspective seeks to answer two questions: one empirical and one normative. The empirical question, “What is current neuroscience journal guidance with respect to reporting race and ethnicity population descriptors, and what variation exists across journals?”, has a clear answer: Currently, the guidance for reporting race and ethnicity population descriptors is not consistent across neuroscience journals. The normative question is more challenging: *should* such guidance be standardized, and if so, what should those standards be?

In answering this question, it is important to differentiate between procedural and substantive standardization. *Procedural* standards refer to the steps that researchers need to take (e.g., requiring every researcher to report on their decision about whether to include race and if so, how they defined it). *Substantive* standards refer to the actual substance of the variables (e.g., requiring every study to define race the same way).

Given the recognized variation in how individuals define race and ethnicity, it is not advisable for journals to collectively institute a universal standard for measuring these descriptors. We agree with Cardenas-Iniguez and Gonzalez (2024) that “no categorical definition of race and ethnicity is exhaustive and without limitations,” and “researchers should be intentional, equitable and responsible for their selected operationalization of race and ethnicity” (Cardenas-Iniguez and Gonzalez, 2024). Reporting clear and precise definitions to readers will facilitate reproducibility, generalizability, and meta-analyses. Moreover, without clarity on how race and ethnicity variables are constructed, it is difficult to assess whether the field is making progress on its participant diversity goals. Although researchers need not wait for journal guidance to adopt these reporting practices, journal editors and publishing organizations function as gatekeepers with influence on whether and how population demographics data are reported. We suggest that the policy should require a statement as to why and how race and ethnicity population descriptors are (or are not) defined, collected, analyzed, and reported in a given study.

As noted, debate persists about whether and when utilizing race as a variable is appropriate, particularly given the documented history of population descriptors being misused to forge scientific validity to prejudicial beliefs (Gee and Ford, 2011; Roy, 2018; McIntire 2026). In the nineteenth and early twentieth centuries, practices such as cranial measurement were employed to rank intelligence across racialized groups, reinforcing hierarchical and biologically deterministic interpretations of human variation (Mitchell, 2018). Although such claims have been discredited, their methodological legacy persists when socially constructed categories are treated as proxies for biological difference in contemporary neuroimaging analyses (Ricard et al., 2023). These legacies continue to shape study design, sampling strategies, and interpretation, including the conflation of social vulnerability with biological risk (Rebello and Uban, 2023). Without explicit justification and careful interpretation, group-level comparisons risk reintroducing outdated assumptions under modern analytic frameworks, particularly when race or ethnicity is implicitly framed as biologically meaningful rather than as reflecting social, environmental, and structural systems. Therefore, it is important to question not only the scientific justification for reporting race, ethnicity, and/or ancestry, but also the ethical implications (Boyd et al., 2020; Brothers et al., 2021; Brothers and Cho, 2021).

The socially negotiated nature of race and ethnicity, alongside the subjective nature of self-identification, make standardizing the definitions of these variables a challenging and likely impossible task. Researchers will define the variables differently, and some researchers may jettison these population descriptors altogether. Following the guidance of AMA and *Nature*, our favored approach, emphasizing procedural standards, would allow for such decisions. By requiring the research team to clarify the reasons for inclusion or exclusion of race and ethnicity and their definitions, research findings can better contribute toward efforts to combat health inequities in the sciences. We note that sometimes the explanations will be straightforward. For instance, in secondary analyses the variables might be excluded because they were never collected in the original dataset, and the variables will be defined in whatever ways the original dataset defined them.

### Examples of best practices from major consortia and adjacent fields

Several large-scale neuroimaging and biomedical consortia have adopted rigorous and ethically informed approaches regarding the use of race, ethnicity, and ancestry descriptors, serving as useful models for the neuroscientific community. The Adolescent Brain Cognitive Development (ABCD) Study cautions against the misuse of population descriptors in genetic analyses and emphasizes responsible interpretation as a condition of data access (Dick et al., 2021; Saragosa-Harris et al., 2022). The ABCD notes that self-reported race and ethnicity primarily reflect social and environmental experiences rather than genetic variation (Saragosa-Harris et al., 2022; Dick et al., 2021). Accordingly, researchers should exercise caution when including race and/or ethnicity as a covariate, as doing so implicitly carries assumptions about what such variables do and do not measure, masking underlying confounds. This approach aligns with genomics best practices (as advised by the NASEM) by distinguishing socially defined variables from genetically inferred measures and reducing the risk of conflating social categories with biological mechanisms while still supporting population-level analyses (National Academies of Sciences, Engineering, and Medicine, 2023).

Similarly, the United Kingdom (UK) Biobank demonstrates transparent demographic reporting and explicit acknowledgment of structural limitations in cohort composition (UK Biobank, 2025; UK Biobank, n.d.). The UK Biobank employs standardized ethnic background categories with defined subcategories, (UK Biobank, n.d.) while publishing clear statements regarding selection biases, including “healthy volunteer bias” and socioeconomic skew (UK Biobank, 2025). By documenting these limitations and discussing their implications for generalizability, the UK Biobank provides researchers with context for interpretation rather than implying global representativeness. Adjacent fields, including genetics and epidemiology, have articulated recommendations through professional societies and research programs such as the National Heart, Lung, and Blood Institute Trans-Omics for Precision Medicine initiative, and the International Genetic Epidemiology Society. (Khan et al., 2022; International Genetic Epidemiology Society (n.d.). These recommendations emphasize careful terminology, explicit justification for population groupings, avoidance of using race or ethnicity as proxies for genetic ancestry, preservation of population-specific information, and consideration of potential harms alongside analytic benefits (Khan et al., 2022; International Genetic Epidemiology Society, n.d.). Together, these examples suggest that best practices may not require eliminating population descriptors, but rather their deliberate, transparent, and standardized use—an approach that human neuroimaging research is well-positioned to adopt.

## Recommendations and Conclusion

What strategies are most promising for improving more robust consideration of race and ethnicity population descriptors? We focus on three concrete next steps.

1. *Be transparent about decisions on whether and how race, ethnicity, and ancestry are used in human neuroscience studies.*We have briefly discussed above the ongoing debate about whether and how to include these variables in neuroscience studies. We anticipate that this debate will continue, with well-reasoned arguments on both sides. Thus we advocate for journal guidance that provides researchers with flexibility on these decisions, but that also requires transparency about why race and ethnicity variables are (or are not) utilized and how they are operationalized.
2. *Develop field-wide procedural standards. Neuroscience journal editors should review existing guidelines (e.g., *Nature*, AMA, ICMJE) and assess whether updates are needed.*Ideally, journals and publishers would collaborate to co-create field-wide standards, enabling easier cross-journal comparisons and reducing the burden on researchers. With respect to race and ethnicity population descriptors, journals should promulgate procedural standards with sufficient flexibility to apply to a wide range of human neuroscience studies across the world. To this end, we are pursuing a new project, *Improving Recruitment, Engagement, and Access for Community Health Equity for BRAIN Next-Generation Human Neuroimaging Research and Beyond* (REACH for BRAIN). REACH for BRAIN is utilizing an expert working group and community input to fill the guidance gap on the reporting of race and ethnicity descriptors in neuroscience research. We encourage journal editors and neuroscience researchers to engage with us as we develop this guidance.
3. *Promote compliance by learning lessons from Sex and Gender Equity in Research (SAGER).* The SAGER guidelines were developed over the course of three years, and published in 2016. In 2018, the SAGER Working Group was established to “focus on dissemination, implementation, and monitoring of the SAGER Guidelines and other activities related to mainstreaming gender in research conduct” (European Association of Science Editors, n.d.). After the working group had amassed widespread adoption - notably by the World Health Organization - their activities were delegated to a non-profit, GENDRO (Heidari et al., 2024). The key takeaway from this effort is that establishing reporting guidelines is only a first step and achieving consistent adherence requires extensive and ongoing follow up.

At present, most human neuroscience studies are silent with respect to why race and/or ethnicity were or were not included, and similarly silent about why those variables were defined as they were. Taking these next steps would greatly increase transparency, allowing the neuroscience research community to understand the research sample, and to contextualize the findings in the landscape of the field. For the field, greater transparency and consistency in reporting would improve study interpretability and generalizability because we would know what was actually measured—and why. This precision in variable definition would also promote cross-cohort harmonization and facilitate grater collaboration, replication, and meta-analyses.

For research teams, this standard would require more upfront discussion during study design about the potential inclusion and operationalization of race and ethnicity variables. Such discussion, which we stress should happen for all potentially included population descriptor variables, would likely lead to more thoughtful research designs. Importantly, such discussions would lead researchers to more frequently confront a history in which purported racial differences in brain structure were utilized to perpetuate racism (Poskett, 2019). The ongoing misuse of racial categories in neuroscience, while intertwined with the marginalization of historically minoritized populations, reflects distinct issues: methodological errors rooted in false biological premises, and systemic exclusion that obscures structural determinants such as socioeconomic condition, environmental exposures, and access to care. These legacies continue to shape study design, sampling strategies, and interpretation, including the conflation of social vulnerability with biological risk (Rebello and Uban, 2023). Without explicit justification and careful interpretation, group-level comparisons risk reintroducing outdated assumptions under modern analytic frameworks, particularly when race or ethnicity is implicitly framed as biologically meaningful rather than as reflecting social, environmental, and structural systems.

## Data Availability

The original contributions presented in the study are included in the article/Supplementary material. Further inquiries can be directed to the corresponding author.

## Footnotes

1 Two publishing companies also were contacted: Cell Press and Oxford Academic.

2 For those journals where an initial reply was not received within two and a half weeks, a follow-up email was sent. Non-responsive journals are those that did not reply to either our first or second inquiries.

3 The journals requiring or advising compliance with the Helsinki Declaration are: *Acta Neuropathologica, Annals of Neurology, Alzheimer’s Research & Therapy, Behavioral and Brain Sciences, Brain, Brain Behavior and Immunity, Dialogues in Clinical Neuroscience, Human Brain Mapping, Journal of Neuroinflammation, Journal of Pineal Research, Molecular Psychiatry, Nature Human Behavior, Nature Neuroscience, Nature Reviews Neuroscience, Neuron, Neuroscience and Biobehavioral Reviews, npj Parkinson’s Disease, Oxford Open Neuroscience, Psychiatry and Clinical Neurosciences, and Translational Neurodegeneration.*
