# Supplementary material for: Addressing ethical challenges in the reporting of race and ethnicity population descriptors in human neuroscience research
Source: Front Neuroimaging. 2026 Jul 1;5:1744569. doi: 10.3389/fnimg.2026.1744569 (PMC13388039; doi:10.3389/fnimg.2026.1744569)
Supplement: Supplementary file 1 [file Supplementary_file_1.docx]

*Addressing Ethical Challenges in Reporting of Race and Ethnicity Population Descriptors in Human Neuroscience Research*

**Supplementary Information**

**Supplemental 1: Nature and AMA Guidance on Reporting Race and Ethnicity Population Descriptors**

Guidance from International Committee of Medical Journal Editors (ICMJE)

- “Authors should define how they determined race or ethnicity and justify their relevance. In the case where race or ethnicity was not collected, explain why it was not collected.”
- “Race and ethnicity are social and not biological constructs; authors should interpret results associated with race and ethnicity in that context.”
- “Authors should use neutral, precise, and respectful language to describe study participants and avoid the use of terminology that might stigmatize participants.” Page 17

Guidance from ICMJE’s Uniform Requirements for Manuscripts Submitted to Biomedical Journals: Writing and Editing for Biomedical Publication

- “When authors use variables such as race or ethnicity, they should define how they measured the variables and justify their relevance.”

Guidance from Consolidated Standards of Reporting Trials (CONSORT)

- “Baseline data: a table showing baseline demographics and clinical characteristics for each group”

Guidance from Human Brain Mapping

- "When detailing demographic information about a study population, it is advisable to use terms to designate ethnicity (e.g. African American and South Asian) rather than race. The British Sociological Association (BSA) have devised some language guidelines for when referring to ethnicity and race."
- References, but does not mandate ICMJE

Guidance from Dialogues in Clinical Neuroscience

- “Human studies categorized by such groupings should include an explanation of the definitions and categories, including whether any rules of human categorization were required by the relevant funding agencies."
- Follows ICMJE for all relevant research

Guidance from Nature

Conceptual Framework:

- “Race and ethnicity are sociopolitical constructs. Humans do not have biological races, at least based on modern biological criteria for the identification of geographical races or subspecies.”

Comprehensive Race and Ethnicity Reporting:

- “For studies involving humans categorized by race, ethnicity, national or social origin, sex, gender identity, sexual orientation, religion, political or other beliefs, age, disease, (dis)ability, socio-economic status, or other socially constructed or socially relevant groupings, authors should:
  - “Explicitly describe their methods of categorizing human populations
  - “Define categories in as much detail as the study protocol allows
  - “Justify their choices of definitions and categories, including for example whether any rules of categorization were required by their funding agency
  - “Explain whether (and if so, how) they controlled for confounding variables in their analyses”
- “Studies that use the constructs of race and/or ethnicity should explicitly motivate their use. Race/ethnicity should not be used as proxies for other variables – for example, socioeconomic status or income. For studies involving data collected from human participants, researchers should explain:
  - “who provided the classification terms (the participants, the researchers or third parties);
  - “what the classification terms are;
  - “how racial/ethnic identity was determined (by the participants, the researchers or third parties).
  - “This information should be provided in the Methods section of your manuscript and in the Reporting Summary.“

Inclusive, Specific, and Respectful Language:

- “we require that all content submitted for publication be respectful of the dignity and rights of individuals and human groups. Researchers are asked to carefully consider the potential implications (including inadvertent consequences) of research on human groups defined by attributes of race, ethnicity, national or social origin, sex, gender identity, sexual orientation, religion, political or other beliefs, age, disease, (dis)ability or other status, to be reflective of their authorial perspective if not part of the group under study, and contextualise their findings to minimize as much as possible potential misuse or risks of harm to the studied groups in the public sphere.“
- “authors should use inclusive, respectful, non-stigmatizing language in their submitted manuscripts. Authors should ensure that writing is free from stereotypes or cultural assumptions. We recommend avoiding the use of descriptors that refer to attributes such as race, ethnicity, national or social origin, sex, gender identity, sexual orientation, religion, political or other beliefs, age, disease, (dis)ability or other group descriptors unless they are relevant. We advise that authors writing in English follow the guidance on bias-free language provided by the American Psychological Association when preparing their manuscripts for submission.“

Revision or Rejection on the Basis of Harmful Content:

- “Racism is scientifically unfounded and ethically untenable. Editors reserve the right to request modifications to (or correct or otherwise amend post-publication), and in severe cases refuse publication of (or retract post-publication), racist content. Editors use the guiding criteria I-IV set out in the section Research on human populations (see above) to identify content that potentially undermines the equal dignity and rights of humans of all races/ethnicities. "
- “Regardless of content type (research, review or opinion) and, for research, regardless of whether a research project was reviewed and approved by an appropriate institutional ethics committee, editors reserve the right to request modifications to (or correct or otherwise amend post-publication), and in severe cases refuse publication of (or retract post-publication):
  - “Content that is premised upon the assumption of inherent biological, social, or cultural superiority or inferiority of one human group over another based on race, ethnicity, national or social origin, sex, gender identity, sexual orientation, religion, political or other beliefs, age, disease, (dis)ability, or other socially constructed or socially relevant groupings (hereafter referred to as socially constructed or socially relevant human groupings).
  - “Content that undermines - or could reasonably be perceived to undermine - the rights and dignities of an individual or human group on the basis of socially constructed or socially relevant human groupings.
  - “Content that includes text or images that directly or indirectly disparage a person or group on the basis of socially constructed or socially relevant human groupings.
  - “Submissions that embody singular, privileged perspectives, which are exclusionary of a diversity of voices in relation to socially constructed or socially relevant human groupings, and which purport such perspectives to be generalisable and/or assumed.”

Consideration of Genetic Ancestry:

- “Biomedical studies should not conflate genetic ancestry (a biological construct) and race/ethnicity (sociopolitical constructs): although race/ethnicity are important constructs for the study of disparities in health outcomes and health care, empirically established genetic ancestry is the appropriate construct for the study of the biological aetiology of diseases or differences in treatment response. If race/ethnicity are used in the context of disease aetiology due to the unavailability of genetic ancestry data, this should be done with caution and clarification. “

Guidance from American Medical Association (AMA)

Conceptual Framework:

- “This guidance continues to acknowledge that race and ethnicity are social constructs as well as the important sensitivities and controversies related to use of these terms and associated nomenclature in medical and health research, education, and practice. Thus, for content published in medical journals, language and terminology must be accurate, clear, and precise, and must reflect fairness, equity, and consistency in use and reporting of race and ethnicity. The guidance also acknowledges that the reporting of race and ethnicity should not be considered in isolation and should be accompanied by reporting of other sociodemographic factors and social determinants, including concerns about racism, disparities, and inequities, and the intersectionality of race and ethnicity with these other factors.”

Comprehensive Race and Ethnicity Reporting:

- “The Methods section should include an explanation of who identified participant race and ethnicity and the source of the classifications used (eg, self-report or selection, investigator observed, database, electronic health record, survey instrument).”
- “If race and ethnicity categories were collected for a study, the reasons that these were assessed also should be described in the Methods section. If collection of data on race and ethnicity was required by the funding agency, that should be noted.”
- “Specific racial and ethnic categories are preferred over collective terms, when possible. Authors should report the specific categories used in their studies and recognize that these categories will differ based on the databases or surveys used, the requirements of funders, and the geographic location of data collection or study participants. Categories included in groups labeled as “other” should be defined.”
- “Categories should be listed in alphabetical order in text and tables.”
- “Race and ethnicity categories of the study population should be reported in the Results section.”

Inclusive, Specific, and Respectful Language:

- “Specific racial and ethnic terms are preferred over collective terms, when possible.”
- “Racial and ethnic terms should not be used in noun form (eg, avoid Asians, Blacks, Hispanics, or Whites); the adjectival form is preferred (eg, Asian women, Black patients, Hispanic children, or White participants) because this follows AMA style regarding person-first language.”
- “In agreement with other guides,13,24 other terms related to colors, such as brown and yellow, should not be used to describe individuals or groups. These terms may be less inclusive than intended or considered pejorative or a racial slur.”
- “ the term people of color was introduced to mean all racial and ethnic groups that are not considered White or of European ancestry and as an indication of antiracist, multiracial solidarity. However, there is concern that the term may be “too inclusive,” to the point that it erases differences among specific groups.13,26-28 There are similar concerns about use of the collective and abbreviated terms for Black, Indigenous, and people of color (BIPOC) and Black, Asian, and minority ethnic (BAME) (commonly used in the UK).”
- “When collective terms are used, merging of race and ethnicity with a virgule as “race/ethnicity” is no longer recommended. Instead, “race and ethnicity” is preferred, with the understanding that there are numerous subcategories within race and ethnicity.”
- “The terms African American or Black may be used to describe participants in studies involving populations in the US, following how such information was recorded or collected for the study. However, the 2 terms should not be used interchangeably in reports of research unless both terms were formally used in the study, and the terms should be used consistently within a specific article. For example, among Black people residing in the US, those from the Caribbean may identify as Black but not as African American, whereas Black people whose families have been in the US for several generations may identify as Black and African American. “
- “In reference to persons indigenous to North America (and their descendants), American Indian or Alaska Native is generally preferred to the broader term Native American.”
- “ it is generally preferable to describe persons of Asian ancestry according to their country or regional area of origin (eg, Cambodian, Chinese, Indian, Japanese, Korean, Sri Lankan, East Asian, Southeast Asian).”
- “the term Caucasian had historically been used to indicate the term White, but it is technically specific to people from the Caucasus region in Eurasia and thus should not be used except when referring to people from this region.”
- “Hispanic, Latino or Latina, Latinx, and Latine are terms that have been used for people living in the US of Spanish-speaking or Latin American descent or heritage, but as with other terms, they can include people from other geographic locations.29,30 Hispanic historically has been associated with people from Spain or other Spanish-speaking countries in the Western hemisphere (eg, Cuba, Central and South America, Mexico, Puerto Rico); however, individuals and some government agencies may prefer to specify country of origin.29-31 Latino or Latina are broad terms that have been used for people of origin or descent from Cuba, Mexico, Puerto Rico, and some countries in Central America, South America, and the Caribbean, but again, individuals may prefer to specify their country of origin.29-31 When possible, a more specific term (eg, Cuban, Cuban American, Guatemalan, Latin American, Mexican, Mexican American, Puerto Rican) should be used.”
- The general term minorities should not be used when describing groups or populations because it is overly vague and implies a hierarchy among groups. Instead, include a modifier when using the word “minority” and do not use the term as a stand-alone noun, for example, racial and ethnic minority groups and racial and ethnic minority individuals.11,24 However, even this umbrella term may not be appropriate in some settings. Other terms such as underserved populations (eg, when referring to health disparities among groups) or underrepresented populations (eg, when referring to a disproportionately low number of individuals in a workforce or educational program) may be used provided the categories of individuals included are defined at first mention.

Overall Demographic Reporting:

- “Aggregate, deidentified demographic information (eg, age, sex, race and ethnicity, and socioeconomic indicators) should be reported for research reports along with all prespecified outcomes”
- “Demographic variables collected for a specific study should be indicated in the Methods section. Demographic information assessed should be reported in the Results section, either in the main article or in an online supplement or both. “
- “If any demographic characteristics that were collected are not reported, the reason should be stated.”

Nuanced Categorization:

- “Researchers should aim for inclusivity by providing comprehensive categories and subcategories where applicable. Many people may identify with more than 1 race and ethnicity; therefore, categories should not be considered absolute or viewed in isolation.”
- “If the criteria for data quality and confidentiality are met, at a minimum, the number of individuals identifying with more than 1 race should be reported. Authors are encouraged to provide greater detail about the distribution of multiple racial and ethnic categories if known. In general, the term mixed race may carry negative connotations13 and should be avoided, unless it was specifically used in data collection; in this case, the term should be defined, if possible. To the extent possible, the specific type of multiracial and multiethnic groups should be delineated.”

Privacy and Ethical Considerations:

- “ If the numbers in some categories are so small as to potentially identify study participants, the specific numbers and percentages do not need to be reported provided this is noted. “

Generalizability and Limitations:

- “Use caution in interpreting or generalizing findings from studies of risk based on populations of individuals representing specific or limited racial and ethnic categories.”
- “When reporting the results of research that includes racial and ethnic disparities and inequities, authors are encouraged to provide a balanced, evidence-based discussion of the implications of the findings for addressing institutional racism and structural racism as these affect the study population, disease or disorder studied, and the relevant health care systems. For example, Introduction and Discussion sections of manuscripts could include implications of historical injustices when describing the differences observed by race and ethnicity. Such discussion of implications can use specific words, such as racism, structural racism, racial equity, or racial inequity, when appropriate.”

**Supplemental 2: List of Articles Reviewed in *Biological Psychiatry*, alphabetical order by first author last name**

As discussed in the main text, we reviewed neuroscience studies involving human participants published in *Biological Psychiatry* from February 1, 2024 through August 15, 2024. This Supplementary Information file lists each of the articles we reviewed, and our notes on how the articles reported on race, ethnicity, and ancestry population descriptors.

- Akkouh, I. A., et al. Longitudinal transcriptomic analysis of human cortical spheroids identifies axonal dysregulation in the prenatal brain as a mediator of genetic risk for schizophrenia. *Biological Psychiatry*, **95,** 687-698 (2024).
  - Utilized 3 race categories: African, European, Mixed
- Amminger, G., et al. The Addition of Fish Oil to Cognitive Behavioral Case Management for Youth Depression: A Randomized, Double-Blind, Placebo-Controlled, Multicenter Clinical Trial. *Biological Psychiatry*. **95,** 426-433 (2024).
  - Did not report race or ethnicity population descriptors for participants
- Bach, P., et al. Stress-induced sensitization of insula activation predicts alcohol craving and alcohol use in alcohol use disorder. *Biological Psychiatry*. **95,** 245-255 (2024).
  - Did not report race or ethnicity population descriptors for participants
- Baller, E., et al. Mapping the relationship of white matter lesions to depression in multiple sclerosis. *Biological Psychiatry*. **95,** 1072-1080 (2024).
  - Utilized 9 race and ethnicity categories: American Indian, Asian, Black, East Indian, Hispanic/Latino/Black, Hispanic/Latino/White, Other, Unknown, White
- Bhatt, R., et al. Mapping brain structure variability in chronic pain: The role of widespreadness and pain type and its mediating relationship with suicide attempt. *Biological Psychiatry*. **95,** 473-481 (2024).
  - Utilized 1 race category: White
- Bowler, A., et al. Phenotypic and genetic associations between preschool fine motor skills and later neurodevelopment, psychopathology, and educational achievement. *Biological Psychiatry*. **95,** 849-858 (2024).
  - Utilized 5 race categories: Asian, Black, Mixed Race, White, Other
- Caspi, A., et al. Accelerated pace of aging in schizophrenia: five case-control studies. *Biological Psychiatry*. **95,** 1038-1047 (2024).
  - Utilized 1 race and ethnicity category: White European
  - Utilized 11 ancestry categories: African ancestry, Northern and Western European ancestry, Han Chinese, Chinese, Gujarati Indian, Japanese, Luhya, Mexican, Maasai, Toscani, Yoruba
- Chang, X., Qu, H., Liu, Y., Glessner, J., & Hakonarson, H. A protective role of low polygenic risk score in healthy individuals carrying attention-deficit/hyperactivity disorder–associated copy number variations. *Biological Psychiatry*. **95,** 881-887 (2024).
  - Utilized 2 race categories: European Descent and African-American Ancestry
- Clifton, N., Lin, J., Holt, C., O’Donovan, M., & Mill, J. Enrichment of the local synaptic translatome for genetic risk associated with schizophrenia and autism spectrum disorder. *Biological Psychiatry*. **95,** 888-895 (2024).
  - Utilized 4 ancestry categories: European, East Asian, African American, and Latino
- Crouse, J., et al. Evening Chronotypes With Depression Report Poorer Outcomes of Selective Serotonin Reuptake Inhibitors: A Survey-Based Study of Self-Ratings. *Biological Psychiatry*. **96,** 4-14 (2024).
  - Utilized 1 ancestry category: European
- Davis, B. A., et al. TCF4 mutations disrupt synaptic function through dysregulation of RIMBP2 in patient-derived cortical neurons. *Biological Psychiatry*. **95,** 662-675 (2024).
  - Did not report race or ethnicity population descriptors for participants
- Ding, H., et al. Individualized texture similarity network in schizophrenia. *Biological Psychiatry*. **96,** 176-187 (2024).
  - Did not report race or ethnicity population descriptors for participants
- Gadot, R., et al. Tractography-based modeling explains treatment outcomes in patients undergoing deep brain stimulation for obsessive-compulsive disorder. *Biological Psychiatry*. **96,** 95-100 (2024).
  - Did not report race or ethnicity population descriptors for participants
- Garcia-Argibay, et al. Attention-deficit/hyperactivity disorder and major depressive disorder: Evidence from multiple genetically informed designs. *Biological Psychiatry*. **95,** 444-452 (2024).
  - Did not report race or ethnicity population descriptors for participants
- Ghane, M., et al. Specific Patterns of Endogenous Functional Connectivity Are Associated With Harm Avoidance in Obsessive-Compulsive Disorder. *Biological Psychiatry*. **96,** 137-146 (2024).
  - Did not report race or ethnicity population descriptors for participants
- Gueguen, M., et al. Recent Opioid Use Impedes Range Adaptation in Reinforcement Learning in Human Addiction. *Biological Psychiatry*. **95,** 974-984 (2024).
  - Utilized 6 race and ethnicity categories: Asian, Black or African American, Other or more than one race, White, Hispanic or Latino, Non-Hispanic or Latino
- Halahakoon, D., et al. Pramipexole enhances reward learning by preserving value estimates. *Biological Psychiatry*. **95,** 286-296 (2024).
  - Did not report race or ethnicity population descriptors for participants
- Huang, L., et al. Polygenic Analyses Show Important Differences Between Major Depressive Disorder Symptoms Measured Using Various Instruments. *Biological Psychiatry*. **95,** 1110-1121 (2024).
  - Utilized 1 ancestry category: European
- Iraji, A., et al. Spatial Dynamic Subspaces Encode Sex-Specific Schizophrenia Disruptions in Transient Network Overlap and Their Links to Genetic Risk. *Biological Psychiatry*. **96,** 188-197 (2024).
  - Utilized 4 race categories: White, Black, Asian, Other
- Johnston, K., Cote, A., Hicks, E., Johnson, J., & Huckins, L. Genetically regulated gene expression in the brain associated with chronic pain: Relationships with clinical traits and potential for drug repurposing. *Biological Psychiatry*. **95,** 745-761 (2024).
  - Utilized 4 ancestry categories: East Asian, African American, Southeast Asian, Hispanic American, Native American, European American
- Kiltschewskij, D., et al. Alteration of DNA methylation and epigenetic scores associated with features of schizophrenia and common variant genetic risk. *Biological Psychiatry*. **95,** 647-661 (2024).
  - Did not report race or ethnicity population descriptors for participants
- Kosidou, K., et al. Maternal Steroid Hormone Levels in Early Pregnancy and Autism in the Offspring: A Population-Based, Nested Case-Control Study. *Biological Psychiatry*. **96,** 147-158 (2024).
  - Did not report race or ethnicity population descriptors for participants
- Jefsen, O. H., et al. Polygenic risk of mental disorders and subject-specific school grades. *Biological Psychiatry*. **96,** 222-229 (2024).
  - Utilized 1 ancestry category: European
- Li, J., et al. Transcriptomic similarity informs neuromorphic deviations in depression biotypes. *Biological Psychiatry*. **96,** 414-425 (2024).
  - Did not report race or ethnicity population descriptors for participants
- Li, Z., et al. Irritable Bowel Syndrome Is Associated With Brain Health by Neuroimaging, Behavioral, Biochemical, and Genetic Analyses. *Biological Psychiatry*. **96,** 1122-1132 (2024).
  - Utilized 1 race category: White
- Ma, S., et al. A machine learning analysis of big metabolomics data for classifying depression: Model development and validation. *Biological Psychiatry*. **96,** 44-56 (2024).
  - Utilized 1 race category: White
- Mariano, M., Rossetti, I., Maravita, A., Paulesu, E., & Zapparoli, L. Sensory Attenuation Deficit and Auditory Hallucinations in Schizophrenia: A Causal Mechanism or a Risk Factor? Evidence From Meta-Analyses on the N1 Event-Related Potential Component. *Biological Psychiatry*. **96,** 207-221 (2024).
  - Did not report race or ethnicity population descriptors for participants
- Martin, Z., et al. Exaggerated peripheral and systemic vasoconstriction during trauma recall in posttraumatic stress disorder: A co-twin control study. *Biological Psychiatry*. **96,** 278-286 (2024).
  - Did not report race or ethnicity population descriptors for participants
- Maximo, J., Armstrong, W., Kraguljac, N., & Lahti, A. Higher-Order Intrinsic Brain Network Trajectories After Antipsychotic Treatment in Medication-Naïve Patients with First-Episode Psychosis. *Biological Psychiatry*. **96,** 198-206 (2024).
  - Did not report race or ethnicity population descriptors for participants
- McCall, A., et al. Evidence for Locus Coeruleus–Norepinephrine System Abnormality in Military Posttraumatic Stress Disorder Revealed by Neuromelanin-Sensitive Magnetic Resonance Imaging. *Biological Psychiatry*. **96,** 268-277 (2024).
  - Did not report race or ethnicity population descriptors for participants
- Meyer, G., et al. Deep brain stimulation for obsessive-compulsive disorder: optimal stimulation sites. *Biological Psychiatry.* **96,** 101-113 (2024).
  - Did not report race or ethnicity population descriptors for participants
- Miller, A., et al. Neural markers of emotion reactivity and regulation before and after a targeted social rejection: differences among girls with and without suicidal ideation and behavior histories. *Biological Psychiatry*. **95,** 1100-1109 (2024).
  - Utilized 5 race categories: American Indian or Alaska Native, Asian, Black, Hispanic/Latina, White, or more than one race/other
- Minichino, A., et al. Psycho-Pharmacomicrobiomics: A Systematic Review and Meta-analysis. *Biological Psychiatry*. **95,** 611-628 (2024).
  - Did not report race or ethnicity population descriptors for participants
- Mortazavi, L., MacNiven, K., & Knutson, B. Blunted neurobehavioral loss anticipation predicts relapse to stimulant drug use. *Biological Psychiatry*. **95,** 256-265
  - Utilized 6 race categories: Race: Asian, African-American, Caucasian, Hispanic, Multi, Decline to state.
- Nishat, E., Scratch, S., Ameis, S., & Wheeler, A. Disrupted maturation of white matter microstructure after concussion is associated with internalizing behavior scores in female children. *Biological Psychiatry*. **96,** 300-308 (2024).
  - Utilized 7 race and ethnicity categories: Asian, Hispanic, Non-Hispanic Black, Non-Hispanic White, Other/Multi-racial
- Nothdurfter, D., Jawinski, P., & Markett, S. White Matter Tract Integrity Is Reduced in Depression and in Individuals With Genetic Liability to Depression. *Biological Psychiatry*. **95,** 1063-1071 (2024).
  - Did not report race or ethnicity population descriptors for participants
- Onwordi, E. C., et al. Synaptic terminal density early in the course of schizophrenia: An in vivo UCB-J positron emission tomographic imaging study of SV2A. *Biological Psychiatry*. **95,** 639-646 (2024).
  - Utilized 4 race categories: Asian, Black, Other, White
- Petrican, R., Fornito, A., & Boyland, E. Lifestyle Factors Counteract the Neurodevelopmental Impact of Genetic Risk for Accelerated Brain Aging in Adolescence. *Biological Psychiatry*. **95,** 453-464 (2024).
  - Utilized 1 race category: Caucasian
- Pfefferbaum, A., et al. Age-accelerated increase of white matter hyperintensity volumes is exacerbated by heavy alcohol use in people living with HIV. *Biological Psychiatry*. **95,** 231-244 (2024).
  - Utilized 4 race categories: Asian, Black, Other, White
- Shan, X., et al. Disentangling the individual-shared and individual-specific subspace of altered brain functional connectivity in autism spectrum disorder. *Biological Psychiatry*. **95,** 870-880 (2024).
  - Did not report race or ethnicity population descriptors for participants
- Shao, J., et al. Capturing the Individual Deviations From Normative Models of Brain Structure for Depression Diagnosis and Treatment. *Biological Psychiatry*. **95,** 403-413 (2024).
  - Did not report race or ethnicity population descriptors for participants
- Sen, P., et al. Dysregulation of microbiota in patients with first-episode psychosis is associated with symptom severity and treatment response. *Biological Psychiatry*. **95,** 370-379 (2024).
  - Did not report race or ethnicity population descriptors for participants
- Sheridan, S., et al. Loss of function in the neurodevelopmental disease and schizophrenia-associated gene CYFIP1 in human microglia-like cells supports a functional role in synaptic engulfment. *Biological Psychiatry*. **95,** 676-686 (2024).
  - Did not report race or ethnicity population descriptors for participants
- Solberg, B. S., et al. Maternal fiber intake during pregnancy and development of attention-deficit/hyperactivity disorder symptoms across childhood: The Norwegian Mother, Father, and Child Cohort Study. *Biological Psychiatry*. **95,** 839-848 (2024).
  - Did not report race or ethnicity population descriptors for participants
- Song, Y., et al. Association between taurine level in the hippocampus and major depressive disorder in young women: A proton magnetic resonance spectroscopy study at 7T. *Biological Psychiatry*. **95,** 465-472 (2024).
  - Did not report race or ethnicity population descriptors for participants
- Stein, F., et al. Brain structural network connectivity of formal thought disorder dimensions in affective and psychotic disorders. *Biological Psychiatry*. **95,** 629-638 (2024).
  - Did not report race or ethnicity population descriptors for participants
- Stoliker, D., et al. Neural mechanisms of resting-state networks and the amygdala underlying the cognitive and emotional effects of psilocybin. *Biological Psychiatry*. **96,** 57-66 (2024).
  - Did not report race or ethnicity population descriptors for participants
- Taylor, C., et al. Amplification of positivity treatment for anxiety and depression: A randomized experimental therapeutics trial targeting social reward sensitivity to enhance social connectedness. *Biological Psychiatry*. **95,** 434-443 (2024).
  - Utilized 8 race and ethnicity categories: Asian, black, more than one race, pacific islander, white, unknown/declined to respond, Hispanic, non-Hispanic
- Tsetsos, F., et al. Genome-wide association study points to novel locus for gilles de la tourette syndrome. *Biological Psychiatry*. **96,** 114-124 (2024).
  - Utilized 1 ancestry category: European
- Urso, D., et al. Nucleus basalis of Meynert degeneration predicts cognitive decline in corticobasal syndrome. *Biological Psychiatry*. **95,** 1048-1054 (2024).
  - Did not report race or ethnicity population descriptors for participants
- Verhoef, E., et al. Genome-wide analyses of vocabulary size in infancy and toddlerhood: Associations with attention-deficit/hyperactivity disorder, literacy, and cognition-related traits. *Biological Psychiatry*. **95,** 859-869 (2024).
  - Did not report race or ethnicity population descriptors for participants
- Wang, J., et al. Habenula volume and functional connectivity changes following laparoscopic sleeve gastrectomy for obesity treatment. *Biological Psychiatry*. **95,** 916-925 (2024).
  - Did not report race or ethnicity population descriptors for participants
- Wilkerson, M., et al. Uncommon protein-coding variants associated with suicide attempt in a diverse sample of US Army soldiers. *Biological Psychiatry*. **96,** 15-25 (2024).
  - Utilized 5 race and ethnicity categories: Asian, Black, Hispanic, Other, White
- Xiao, X., et al. Brain functional connectome defines a transdiagnostic dimension shared by cognitive function and psychopathology in preadolescents. *Biological Psychiatry*. **95,** 1081-1090 (2024).
  - Utilized 5 race categories: White, Hispanic, Black, Unknown, Asian
- Yan, W., et al. A brainwide risk score for psychiatric disorder evaluated in a large adolescent population reveals increased divergence among higher-risk groups relative to control participants. *Biological Psychiatry*. **95,** 699-708 (2024).
  - Utilized 1 ancestry category: White
- Zanoaga, M., et al. Brainwide mendelian randomization study of anxiety disorders and symptoms. *Biological Psychiatry*. **95,** 810-817 (2024).
  - Did not report race or ethnicity population descriptors for participants
- Zeng, L., et al. A single-nucleus transcriptome-wide Association Study implicates novel genes in Depression Pathogenesis. *Biological Psychiatry*. **96,** 34-43 (2024).
  - Utilized 1 race category: Non-Latino White
- Zhang, X., Xu, R., Ma, H., Qian, Y., & Zhu, J. Brain structural and functional damage network localization of suicide. *Biological Psychiatry*. **95,** 1091-1099 (2024).
  - Did not report race or ethnicity population descriptors for participants
- Zhao, H., et al. Comparisons of accelerated continuous and intermittent theta burst stimulation for treatment-resistant depression and suicidal Ideation. *Biological Psychiatry*. **96,** 26-33 (2024).
  - Utilized 1 race category: Han Chinese
- Zhi, D., et al. Triple interactions between the environment, brain, and behavior in children: An ABCD study. *Biological Psychiatry*. **95,** 828-838 (2024).
  - Utilized 5 race categories: Black, White, Hispanic, Asian, Other
- Zika, O., et al. Reduction of Aversive Learning Rates in Pavlovian Conditioning by Angiotensin II Antagonist Losartan: A Randomized Controlled Trial. *Biological Psychiatry*. **96,** 247-255 (2024).
  - Did not report race or ethnicity population descriptors for participants
